# Post-traumatic stress symptoms among an Egyptian sample of post-remission COVID-19 survivors: prevalence and sociodemographic and clinical correlates

**DOI:** 10.1186/s43045-021-00102-y

**Published:** 2021-03-29

**Authors:** Mohamed Abdelghani, Mervat S. Hassan, Maha E. Alsadik, Ahmed A. Abdelmoaty, Amira Said, Samar A. Atwa

**Affiliations:** 1 Psychiatry Department, Faculty of Medicine, Zagazig University, PO Box 44519, Zagazig, Egypt; 2 2015-2016 Hubert H. Humphrey Fellowship, Department of Psychology, Virginia Commonwealth University, Richmond, USA; 3 Department of Chest Diseases, College of Medicine, Zagazig University, Zagazig, Egypt; 4 Department of Tropical Medicine, College of Medicine, Zagazig University, Zagazig, Egypt; 5 Department of Anesthesia and Surgical Intensive Care, College of Medicine, Zagazig University, Zagazig, Egypt

**Keywords:** COVID-19 survivors, Post-traumatic stress symptoms, Egypt

## Abstract

**Background:**

Given its global spread, the COVID-19 virus infection itself may be experienced as a traumatic and stressful event among survivors. The post-traumatic stress symptoms (PTSS) among those surviving the disease were under evaluated. This study aimed to identify and compare PTSS and associated correlates among COVID-19 survivors and control subjects. A cross-sectional design with a convenience sampling included a total of 85 adults who survived COVID-19 virus infection and 85 control subjects (matched for age, sex, education, and socioeconomic level) who were recruited from Zagazig University Hospitals, Sharkia Province, Egypt. The participants were interviewed using a semistructured demographic and clinical checklist, Structured Clinical Interview for DSM-5 Axis I Disorders (SCID-5), the Impact of Event Scale-Revised (IES-R), and the Hospital Anxiety Depression Scale (HADS).

**Results:**

Approximately, 72% of COVID-19 survivors experienced moderate-to-severe PTSS (compared to 53% of control subjects). Individuals who survived the COVID-19 virus infection were more likely to have intensified hyperarousal symptoms (OR: 2.7, 95% CI: 1.7–4.4), with higher total IES-R scoring (OR: 1.03, 95% CI: 1.01–1.05). Among COVID-19 survivors, those who reported moderate-to-severe PTSS were likely to experience severe COVID-19 symptoms during their illness (OR: 4.1, 95% CI: 1.4–11.9).

**Conclusions:**

PTSS was prevalent among COVID-19 survivors in Egypt. The hyperarousal symptoms were the most experienced ones. The symptom severity of COVID-19 virus infection predicted PTSS in COVID-19 survivors.

## Background

Patients who experience post-traumatic stress symptoms (PTSS) live under the shadow of past traumatic accidents. According to the Diagnostic and Statistics of Mental Disorders, the fifth edition (DSM-5), the symptoms include persistence of intrusive thoughts, avoidance of trauma-related cues, and dysregulation in mood and cognitive functions with obvious changes in arousal level. These symptoms are associated and complicated with interpersonal and occupational dysfunctions [1]. In December 2019, a novel coronavirus disease (COVID-19) emerged in Wuhan, China, and by May 18, 2020, the outbreak has catastrophically spread resulting in around 5 million confirmed cases of COVID-19 affections worldwide [2]. With the increased COVID-19 related morbidity and mortality, individuals started to experience extensive illness-related fears, and higher levels of anxiety and depression, with increased susceptibility to physical and psychological distress [3, 4].

The nature and severity of the traumatic event are considered the most robust predictor of PTSS during and after an epidemic. Most epidemiological studies stated that the survivors, followed by their families and health care providers, were more likely to experience PTSS [5]. Survivors of severe COVID-19 symptoms frequently suffer, during their illness, from intense respiratory symptoms which may progress to respiratory failure [6]. Moreover, patients may be subject to physical pain from interventions such as endotracheal intubation, and extreme stressors owing to fear of death from a fatal illness, inability to communicate, and feelings of loss of control. Survivors may be also traumatized after witnessing the death of their family members [7–9]. Therefore, surviving COVID-19 illness would represent near-death or fatal illness experiences, that would be sufficient to develop PTSS.

It is, also, claimed that most individuals diagnosed with COVID-19 have to isolate themselves, which is associated with the development of various psychological symptoms, including PTSS [10]. There are various stressors associated with isolation, which are associated with increased risk of developing psychological disturbances: (i) apprehensions about own health or fears of infecting their families; (ii) feelings of frustration, boredom, and a sense of isolation or even rejection from others; (iii) improper or inaccurate information from the official authorities regarding the nature and severity of the pandemic, or the instructions and actions to take; (iv) lack of basic resources like nutrition, clothes, or accommodation; and (v) financial and socioeconomic problems [10, 11].

All these aforementioned factors seem to apply to the current pandemic. Therefore, determining the psychological impact of COVID-19, particularly PTSS, among individuals who survived the outbreak, would be an essential step for implementing early interventions, aiming to reduce the possibility of further irrational behaviors and helping those populations return to their normal life. So, this study aimed to identify and compare the PTSS and their associated correlates among individuals who survived the COVID-19 outbreak and their control counterparts.

## Methods

### Subjects and study design

This study was a comparative cross-sectional study. Data, utilized in this study, were collected by nonprobability convenience sampling method encompassing a total of 85 COVID-19 survivors who were consecutively enrolled according to the WHO discharge criteria adopted by the Zagazig University isolation hospitals, Zagazig City, Egypt. The inclusion criteria for the COVID-19 survivors included adults of both sexes who were previously diagnosed as COVID-19 positive ones according to their presenting symptoms, and their nasopharyngeal swab confirmed the presence of the offending viral nucleic acid. The COVID-19 survivors were interviewed provided that they were recovered from their COVID-19 infection, and at least one month had been elapsed since completeness of their home isolation or being discharged from hospitalization with stabilization of their general medical condition). An equal number of non-COVID-19 control subjects were also recruited. They were matched for age, sex, and educational level. They were first-degree relatives never diagnosed as positive COVID-19 patients and were chosen to differentiate between the shared environmental and genetic factors and the potential COVID-19-related effects on their mental health status. The sample size was calculated using Epi Info 6.0, at 80% power of the study, 95% confidence level [12]. Exclusion criteria for both groups included individuals younger than 18 or older than 60 years, those who had current comorbid physical or psychiatric disorders, illiterate, and those who refused to participate. All data were collected during the period from September 1 to November 29, 2020.

### Data collection and measures

A semi-structured sheet was designed to collect relevant demographic and clinical data from all participants. It encompassed questions about age, gender, marital status, residence, level of education, residence, occupation, and history of psychiatric and medical illnesses.

#### Structured Clinical Interview for DSM-5 Axis I Disorders (SCID-5)

It is a semi-structured interview utilized to help the examiner confirm or exclude the presence of current major primary mental illnesses [13]. Several studies had established the validity and reliability of this procedure [14, 15].

#### Impact of Events Scale-Revised (IES-R)

It is a 22-item self-report tool utilized to measure the subjective distress associated with traumatic events [16]. The overall score was categorized into normal (0–17), PTSD-like symptoms (18–23), and diagnosis of PTSD (above 24). Its three subscales, namely, intrusion, avoidance, and hyperarousal are closely representatives of the PTSD symptoms. A cut-off score of 2 or more, for each subscale, implies a moderate level of distress. An Arabic validated version of the IES-R scale was previously used among Egyptians from various populations (during the COVID-19 outbreak) [17]. In this study, the Arabic version with good validity and reliability was applied [18].

#### Hospital Anxiety and Depression scale (HADS)

Depressive and anxiety symptoms were considered as potential covariates in this study. HADS is a self-report rating scale used for assessing the associated symptoms of anxiety and depression among participants [19]. It is comprised of 14 items (7 items for each subscale) on a 4-point Likert scale calculated from 0 to 3. The overall score for each subscale, representing the sum of each subscale’s 7 items, ranges from 0 to 21. The Arabic version of this scale, utilized in this study, was previously examined for its reliability and validity [20], and previously applied among the Egyptian populations [4].

### Statistical analyses

The collected data were analyzed using the Statistical Package for the Social Sciences software version 16.0. For categorical variables, the chi-square test was used, while the continuous variables were compared using the independent sample *t* test, and a nonparametric test (Mann-Whitney *U*) for data not normally distributed. To obtain odds ratios (OR) and 95% confidence intervals (CI) of PTSS among COVID-19 survivors and control subjects, the logistic regression analysis was used to adjust for associated anxiety and depressive symptoms. All results were statistically significant when their significant probability was less than 5% (*p* < 0.05).

## Results

### Sociodemographic and clinical characteristics of COVID-19 survivors

The mean age of COVID-19 survivors was 36.0 ± 9.4 years. The majority of them were females (81%), of low-to-moderate education (62%), from rural areas (65%), married (88%), and skilled workers (90%). Around one-fifth and one-tenth of COVID-19 survivors reported histories of medical and psychiatric illnesses, respectively. Besides, COVID-19 survivors were subdivided, according to their presenting symptoms during infection, into mild cases with only home isolation (*n* = 40, 47.1%), moderate cases necessitating hospitalization (*n* = 38, 44.7%), and severe cases necessitating ICU admission (*n* = 7, 8.2%). In terms of sociodemographic variables, there were no statistical differences between both groups. However, compared with their control counterparts, COVID-19 survivors were found to experience higher levels of anxiety and depression (*P* value = 0.001, and 0.003, respectively), as displayed in Table 1.

**Table 1:** Sociodemographic and clinical characteristics of study participants

Variable		COVID-19 survivors (***n*** = 85)		Control (***n*** = 85)		***t***	***P*** value
Age	Mean ± SD	36.0 ± 9.40		33.7 ± 9.37		1.58	0.117
		**No**	**%**	**No**	**%**	***χ***^**2**^	
Sex	Female	69	81.2	62	72.9	1.63	0.202
	Male	16	18.2	23	27.1		
Level of education	Low to moderate	53	62.4	48	56.5	0.61	0.435
	High	32	37.6	37	43.5		
Residence	Rural	55	64.7	46	54.1	1.976	0.160
	Urban	30	35.3	39	45.9		
Marital status	Married	75	88.2	66	77.6	3.37	0.066
	Not married	10	11.8	19	22.4		
Occupation	Skilled	77	90.6	70	82.4	2.65	0.266
	Employee	7	8.2	12	14.1		
	Unemployed	1	1.2	3	2.4		
Hx of medical illnesses	Yes	19	22.4	11	12.9	2.590	0.108
	No	66	77.6	74	87.1		
Hx of psychiatric illnesses	Yes	9	10.6	6	7.1	0.083	0.773
	No	76	89.4	79	92.9		
HADS scoring	Mean ± SD					**MWU**	
*Depression*		8.2 ± 4.2		6.6 ± 3.9		−2.95	**0.003**
*Anxiety*		8.1 ± 4.2		6.1 ± 3.4		−3.33	**0.001**

Bold text indicates statistical significance where *P* value < 0.05

### Post-traumatic stress symptoms in COVID-19 survivors

The prevalence of moderate-to-severe PTSD symptoms among COVID-19 survivors is 72% (compared to 53% among the control subjects). Compared to the control group, the COVID-19 survivors had significantly increased levels of all PTSD symptoms. The affected symptoms were avoidance (*P* value = 0.006), intrusion (*P* value = 0.042), hyperarousal (*P* value <0.001), and total IES-R score (*P* value = 0.011), as displayed in Table 2. Even after being adjusted for associated anxiety and depressive symptoms, the COVID-19 survivors, compared to the control subjects, had greater odds of hyperarousal symptoms (*P* value <0.001, OR: 0.3, 95% CI: 0.2–0.5), and total IES-R score (*P* value = 0.015, OR: 1.03, 95% CI: 1.01–1.05), as displayed in Table 3.

**Table 2:** PTSD symptoms among COVID-19 survivors and control subjects

Variable	COVID-19 Survivors (***n*** = 85)	Control (***n*** = 85)	Test	***P*** value
	Mean ± SD			
			**MWU**	
Avoidance	1.80 ± 0.87	1.43 ± 0.93	−2.74	**0.006**
Intrusion	1.68 ± 0.83	1.37 ± 0.94	−2.04	**0.042**
Hyperarousal	2.01 ± 0.90	1.21 ± 0.82	−5.70	**<0.001**
Total mean IES-R	5.48 ± 2.14	4.02 ± 2.33	−4.17	**<0.001**
	***N*** **(%)**		***X***^**2**^	
Total IES-R score				
*No-to-Mild PTSD*	24 (28.2)	40 (47.1)	6.42	**0.011**
*Moderate-to-severe PTSD symptoms*	61 (71.8)	45 (52.9)		

Bold text indicates statistical significance where *P* value < 0.05

**Table 3:** Adjusted logistic regression of COVID-19 survivors and control subjects by PTSD symptoms

Variable	B	S.E.	Wald.	***P*** value	OR	CI (95%)	
Avoidance	0.29	0.19	2.36	0.125	1.34	0.92	1.95
Intrusion	0.16	0.20	0.65	0.421	1.18	0.79	1.74
Hyperarousal	1.01	0.24	17.85	**<0.001**	**2.74**	**1.72**	**4.37**
Total mean IES-R	0.23	0.09	7.35	**0.007**	**1.26**	**1.07**	**1.50**
Total IES-R score	0.03	0.01	5.87	**0.015**	**1.03**	**1.01**	**1.05**

Logistic regression was adjusted for associated depressive and anxiety symptoms. Bold text indicates statistical significance where 95% confidence intervals do not include the null value (1.00)

Among COVID-19 survivors, there were significant associations between moderate-to-severe PTSD symptoms and gender (*P* value = 0.032), level of education (*P* value = 0.014), and history of COVID-19 symptom severity (*P* value = 0.006), as displayed in Table 4. However, following logistic regression analysis, it was found that only the history of COVID-19 symptom severity (i.e., moderate-to-severe COVID-19 symptoms) was associated with moderate-to-severe PTSD symptoms in COVID-19 survivors (*P* value = 0.010, OR: 4.1, 95% CI: 1.4–11.9), as displayed in Table 5.

**Table 4:** Factors associated with PTSD symptoms among COVID-19 survivors

Variable		Total IES-R score				***t***	***P*** value
		No-to-mild symptoms (***n*** = 24)		Moderate-to-severe (***n*** = 61)			
Age	Mean ± SD	34.5 ± 8.5		36.5 ± 9.7		−0.89	0.375
		**No**	**%**	**No**	**%**	***χ***^**2**^	
Gender	Female	16	66.7	53	86.9	4.608	**0.032**
	Male	8	33.3	8	13.1		
Marital status	Married	20	83.3	55	90.2	0.77	0.379
	Not married	4	16.7	6	9.8		
Residence	Rural	15	62.5	40	65.6	0.071	0.790
	Urban	9	37.5	21	34.4		
Level of education	Low to moderate	10	41.7	43	70.5	6.10	**0.014**
	High	14	58.3	18	29.5		
Occupation	Skilled	21	87.5	56	91.8	1.17	0.558
	Employee	3	12.5	4	6.6		
	Unemployed	0	0.0	1	1.6		
Hx of psychiatric illnesses	Yes	3	12.5	4	6.6	0.81	0.370
	No	21	87.5	57	93.4		
Hx of medical illnesses	Yes	5	20.8	14	23.0	0.044	0.833
	No	19	79.2	47	77.0		
Hx of COVID-19 symptom severity	Mild	17	70.8	23	37.7	7.59	**0.006**
	Moderate to severe	7	29.2	38	62.3		
Medications given during COVID-19 infection	Antibiotics	21	95.5	53	94.6	0.02	0.884
	Anticoagulants	13	59.1	36	64.3	0.18	0.669
	Antimalarial	11	50.0	26	46.4	0.08	0.776
	Iverizine	14	63.6	39	69.6	0.26	0.609
	Steroids	10	45.5	35	44.9	1.88	0.170
						**MWU**	
Home isolation (days)	**Mean ± SD**	12.59 ± 10.30		8.56 ± 9.24		−0.91	0.361
Hospital isolation (days)		4.70 ± 6.93		7.25 ± 9.73		−1.04	0.298
HADS scoring							
*Depression*		4.17 ± 2.97		9.72 ± 3.48		−1.41	0.157
*Anxiety*		4.42 ± 2.98		9.74 ± 3.64		−1.93	0.054

Bold text indicates statistical significance where *P* value < 0.05

**Table 5:** Adjusted logistic regression of factors associated with increased PTSD symptoms in COVID-19 survivors

Variable	B	S.E.	Wald.	***P*** value	OR	CI (95%)	
Gender (female)	0.89	0.67	1.75	0.185	2.43	0.65	9.05
Education (lower to moderate)	0.92	0.57	2.62	0.11	2.50	0.83	7.56
Hx of COVID-19 symptom severity (moderate to severe)	1.40	0.55	6.56	**0.010**	**4.07**	**1.39**	**11.92**

Bold text indicates statistical significance where 95% confidence intervals do not include the null value (1.00)

## Discussion

The current study would be one of the few and earliest studies, if any, to present data on the post-discharge mental health consequences of the COVID-19 outbreak among survivors. Data from previous outbreaks, including severe acute respiratory syndrome (SARS) and the Middle East respiratory syndrome (MERS), would explain the increased likelihood of psychiatric symptoms and disorders in COVID-19 survivors [5, 21, 22].

The main finding in this study is that the majority of COVID-19 survivors experienced PTSS. Moreover, compared to the control subjects, the COVID-19 survivors were more likely to suffer from avoidance, intrusion, and hyperarousal symptoms. These findings were consistent with the results from previous outbreak studies. For example, 42% and 27% of MERS survivors experienced symptoms of PTSD at 1 year and 18 months after the outbreak, respectively [23]. Similarly, previous studies found that PTSD symptoms were reported in nearly 39% and 25% of SARS survivors at 12 months and 30 months after discharge, respectively [24, 25].

The results for COVID-19 survivors are much higher than those for control subjects and even for SARS and MERS survivors. These findings would be attributed to several reasons. First, the nature of the COVID-19 virus, being more contagious, with lack of specific antiviral drugs with definite efficacy, and floods of misleading information result in widespread extensive fears and horrors with increased propensity to develop PTSS [26–28]. Second, the separation from family members and close friends is possibly associated with feelings of loneliness and helplessness [29]. Third, the financial burden of the COVID-19 epidemic can add to psychological distress [30].

It would be, however, claimed that the potential neuro-immunological attributes of the COVID-19 virus itself might have additional effects, so that the survivors’ psychological symptoms, as the findings in this study found, would exceed those of normal people sharing the same environmental stressors. It was found that, similar to SARS and MERS patients, the immunological reactions including CNS inflammation, disruption in blood-brain-barrier with the invasion of the peripheral immune cells into the CNS, neurotransmission impairment, hypothalamic-pituitary-adrenal (HPA) axis dysfunction, microglial activation, and indoleamine 2,3 dioxygenase (IDO) release, can biologically explain the psychopathological mechanisms of the associated psychiatric disorders as PSTD among COVID-19 patients [31–33]. Another possible explanation for developing PTSS in COVID-19 survivors is the development of delirium. COVID-19 patients may be at an increased risk for delirium which would be attributed to the neuroinvasive and neurovirulent properties of the virus itself, neuroinflammation, the increased numbers of severe COVID-19 patients who are older adults, and ICU-related causes such as oversedation, prolonged isolation, and interventions like endotracheal intubation and mechanical ventilation [34, 35].

In this study, following logistic regression analysis, the only variable that would predict the severity of PTSS was the history of COVID-19 symptom severity in COVID-19 survivors. Consistent with this finding, Patel and his colleagues found that PTSS had been developed in ICU survivors [36]. In Wuhan, where the COVID-19 outbreak first raised, nearly 3% of COVID-19 patients were intubated with invasive ventilation [37]. Invasive ventilation and prolonged mechanical ventilation were found to be associated with an increased risk for PTSS [38]. Also, it was documented that once the physical illness itself had been more severe, the mental health was more negatively affected [39]. It was claimed that severe complications of the medical illnesses with direct and serious threats to life, with the resultant hospitalization associated with comprehensive medical or invasive interventions to maintain or restore vital functions, would increase the likelihood of developing PTSD symptoms [40, 41].

Finally, despite being lower than that of COVID-19 survivors, the prevalence of moderate-to-severe PTSD symptoms among control subjects, in the current study, was unpredictably high (53%). The control subjects were never hospitalized nor infected with the COVID-19 virus. However, this finding would be explained as those individuals would be subject to a various array of stressors like social restrictions and isolation, financial troubles related to the lockdown regulations, or even worries and fears of being infected themselves or their close relatives and beloved persons, which would have a negative impact on their psychological well-being and quality of life [3, 4].

### Limitations

There are few limitations to this study. First, the COVID-19 survivors were selected from only Zagazig University hospitals. Despite being the largest general hospitals, which were serving the populations in Sharkia Province, but the present findings would not be readily generalized to all COVID-19 survivors of Egypt. Future studies need to include subjects from various governorates so that they will be more representative of the Egyptian population. Second, the main outcome in this study, PTSS, was measured by a self-rating scale with the liability of response bias. Third, it was a cross-sectional study, so interpretation for causality cannot be established. Nonetheless, this study would be one of the earliest studies to reveal the post-remission psychiatric outcomes of COVID-19 survivors in Egypt.

## Conclusions

PTSS was prevalent among COVID-19 survivors in Egypt. The most experienced symptoms were hyperarousal symptoms. The symptom severity of COVID-19 virus infection was the most robust predictor of developing PTSS in COVID-19 survivors. Population-based longitudinal studies are warranted to identify the various neurobiological, immunological, and psychological mechanisms explaining the possible links between PTSS and COVID-19 virus infection, and to investigate the other possible long-term complications among survivors in Egypt.

## Data Availability

The datasets utilized in this study are available from the corresponding author upon reasonable request.

## Abbreviations

DSM-5: Diagnostic and Statistics of Mental Disorders, the fifth edition
HADS: Hospital Anxiety and Depression Scale
IES-R: Impact of Event Scale-Revised
PTSS: Post-traumatic stress symptoms
SCID-5: Structured Clinical Interview for DSM-5 Axis I Disorders
